# Post-Surgical Outcome and Its Determining Factors in Patients Operated on With Focal Cortical Dysplasia Type II—A Retrospective Monocenter Study

**DOI:** 10.3389/fneur.2021.666056

**Published:** 2021-06-09

**Authors:** Attila Rácz, Albert J. Becker, Carlos M. Quesada, Valeri Borger, Hartmut Vatter, Rainer Surges, Christian E. Elger

**Affiliations:** ^1^Department of Epileptology, University Hospital Bonn, Bonn, Germany; ^2^Department of Neuropathology, University Hospital Bonn, Bonn, Germany; ^3^Department of Neurosurgery, University Hospital Bonn, Bonn, Germany

**Keywords:** focal cortical dysplasia, epilepsy surgery, outcome, genetics, Engel classification

## Abstract

**Purpose:** Focal cortical dysplasias (FCDs) are a frequent cause of drug-resistant focal epilepsies. These lesions are in many cases amenable to epilepsy surgery. We examined 12-month and long-term post-surgical outcomes and its predictors including positive family history of epilepsy.

**Methods:** Twelve-month and long-term outcomes regarding seizure control after epilepsy surgery in patients operated on with FCD type II between 2002 and 2019 in the Epilepsy Center of Bonn were evaluated based on patient records and telephone interviews.

**Results:** Overall, 102 patients fulfilled the inclusion criteria. Seventy-one percent of patients at 12 months of follow-up (FU) and 54% of patients at the last available FU (63 ± 5.00 months, median 46.5 months) achieved complete seizure freedom (Engel class IA), and 84 and 69% of patients, respectively, displayed Engel class I outcome. From the examined variables [histopathology: FCD IIA vs. IIB, lobar lesion location: frontal vs. non-frontal, family history for epilepsy, focal to bilateral tonic–clonic seizures (FTBTCS) in case history, completeness of resection, age at epilepsy onset, age at surgery, duration of epilepsy], outcomes at 12 months were determined by interactions of age at onset, duration of epilepsy, age at surgery, extent of resection, and lesion location. Long-term post-surgical outcome was primarily influenced by the extent of resection and history of FTBTCS. Positive family history for epilepsy had a marginal influence on long-term outcomes only.

**Conclusion:** Resective epilepsy surgery in patients with FCD II yields very good outcomes both at 12-month and long-term follow-ups. Complete lesion resection and the absence of FTBTCS prior to surgery are associated with a better outcome.

## Introduction

Focal cortical dysplasias (FCDs) are frequent causes for medically difficult-to-treat epilepsy both in childhood and adulthood (1, 2). FCDs are highly epileptogenic (3, 4), with a distinct pattern of wiring and electrophysiological properties of cellular components (5). Functional alterations of GABAergic and parvalbumin cells have also been demonstrated in surgical specimens from FCDs (6). FCDs are primarily classified according to the Palmini–Taylor system (7), and this system was adopted and extended by the International League Against Epilepsy (ILAE) in 2011 (8). Epilepsy onset with FCD is typically at a young age or childhood (2); however, the age distribution is broad and late onset is not a rare phenomenon either (9). FCDs are primarily localized to the frontal lobe (10–13). Epilepsy surgery often confers seizure freedom to these patients (10–12, 14–22). For post-surgical seizure freedom, the removal of cortical portions of the FCD is necessary and also sufficient, and the removal of subcortical pieces is not a prerequisite (10). Post-surgical outcome regarding seizure control in different FCD subtypes is reported to be different (19, 20, 23, 24). A significant proportion of patients with FCD display positive family history for epilepsy (9), a phenomenon that might be related to genetic factors underlying the formation of FCDs, especially DEPDC5 (25, 26), NPRL2 (27) and NPRL3 (28), and PI3K/AKT (29, 30).

In the Epilepsy Center of Bonn, more than 100 patients were operated on with FCDs during the last 20 years. In this study, we evaluated the case history of these patients by examining their clinical records and focused on long-term outcomes and also evaluated the influence of possible predictors, especially that of family history for epilepsy on post-surgical outcome. In addition, we investigated the possible effect of diagnostic developments on post-surgical outcomes on a longitudinal basis over almost 20 years.

## Methods

We analyzed the clinical charts and records of patients operated on with FCD type II due to their pharmacoresistant epilepsy at the Epilepsy Center of University Hospital Bonn between 2002 and the beginning of 2019 and who also had in- or outpatient visit in our department at least in the context of 12 months post-surgical follow-up (FU). Before surgery, these patients underwent a comprehensive pre-surgical diagnostic workup including brain MRI (1.5 or 3 T field strength), video-EEG monitoring with recording of habitual seizures, neuropsychological evaluation and, in certain cases, MRI post-processing [MAP07 analysis (31, 32)], PET, and ictal/interictal SPECT [SISCOM analysis (33)]. All patients in the reported series had positive MRI; however, in certain cases, several MRI images were needed to delineate the lesion. Patients with dual pathologies were excluded from further analysis. In addition, cases with operation in another center before being evaluated in our department were also discarded. After these inclusion and exclusion criteria, 102 patients were enrolled in the study. In 48 patients (47.06%), further invasive diagnostics was performed, as the non-invasive diagnostics was not conclusive enough. Histopathological confirmation of FCD and further evaluation were performed at the Department of Neuropathology of the University Bonn according to the Palmini–Taylor classification (7). The analyzed patients exclusively had FCD type II, and FCD types I and III were not included. As the ILAE classification (8) also relies on the Palmini–Taylor classification for FCD type II, the ILAE classification and the Palmini–Taylor system give identical results for the current series. Outcomes were primarily extracted from patient charts and letters in our digital database. Patients without any visit in our clinic during the last 12 months preceding study evaluation were contacted in the form of phone interviews after giving written informed consent. Outcomes regarding seizure control were presented according to the Engel classification (34). Some portions and aspects of data (age at epilepsy onset, regional distribution of FCDs, outcomes in a smaller or partially overlapping subset of patients with FCDs) have been analyzed according to different aspects and published elsewhere (9–11). Outcomes at 12 months and at the longest available FU were analyzed separately. For patients operated on twice, outcomes resulting after the first operation were used for the analysis in order to be able to draw conclusions regarding completeness of resection and to avoid further heterogeneities in the patient collective. For statistical analysis, the statistical toolbox of Matlab (MathWorks, Natick) was applied. The influence of possible predictor variables on post-surgical outcome was first tested with univariate analysis, where group comparisons regarding post-surgical outcome [Engel class as the categorical variable and, in a further step, dichotomized outcomes: excellent (Engel I) vs. not excellent (Engel II–IV), favorable (Engel I–II) vs. unfavorable (Engel III–IV) outcome] were performed with Pearson's chi-square statistics and Fisher's exact test. Outcomes at 12 months and at the longest FU for the same patient groups (comparison for the complete series and for patients operated on in different time periods) were compared with the sign test. Afterwards, we applied a multivariate logistic regression analysis with a stepwise regression analysis to verify and to test the influence of a number of possible predictor variables on post-surgical outcome. *p*-values below 0.05 were accepted as an indicator of statistical significance. Charts were generated with the Matlab plotting toolbox and subsequently refined with Paint. The study was carried out after approval from the local ethics committee of the University Hospital Bonn (nr. 126/19). Because of its retrospective nature, informed consent was required only for the phone interviews.

## Results

**Figure 1:**
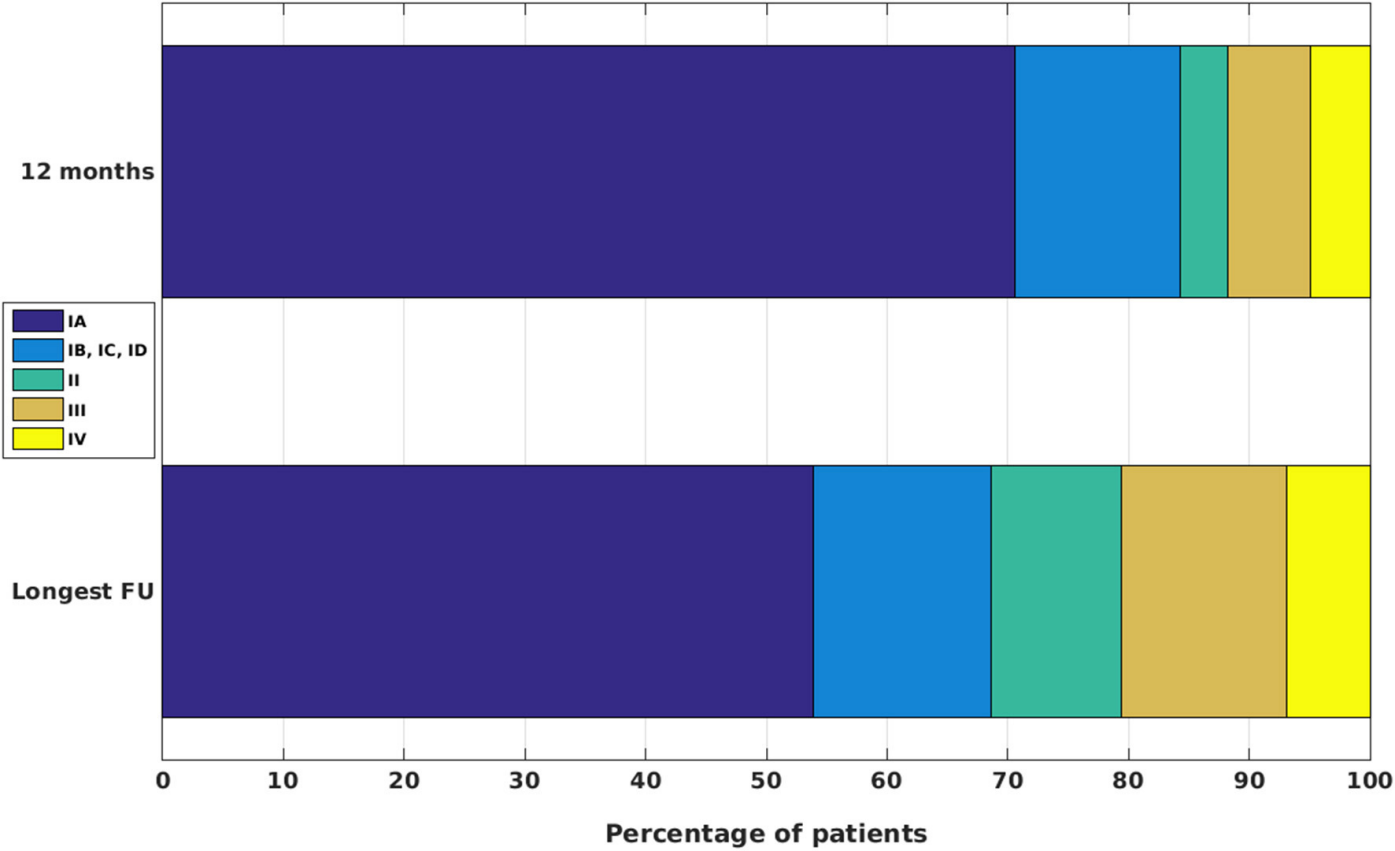
Post-surgical outcomes at 12 months and at the longest available FU for patients with FCD II.

The present analysis comprised 102 patients who had been operated on with histologically verified FCD II between 2002 and the beginning of 2019 in our department. Further characteristics of these patient groups are outlined in Table 1. Histopathological analysis revealed FCD IIA in 22 and FCD IIB in 80 cases. At 12 months of FU, 70.59% of patients were completely seizure free (Engel class IA), and altogether, 84.31% could be categorized as Engel class I [72 patients with IA, 14 patients (13.73%) with IB/IC/ID, 4 patients (3.92%) with Engel class II, 7 (6.86%) with Engel class III, and 5 (4.9%) with Engel class IV outcome; see Figure 1].

**Table 1:** Basic characteristics of patients operated on with medically refractory epilepsy and FCD II.

**Location**	**FCD type IIA**	**FCD type IIB**	**Overall FCD**
**Frontal**	**14**	**51**	**65 (63.73%)**
**Temporal**	**3**	**3**	**6 (5.88%)**
**Parietal**	**2**	**14**	**16 (15.69%)**
**Occipital**	**1**	**2**	**3 (2.94%)**
**Insular**	**1**	**3**	**4 (3.92%)**
**Multilobar**	**1**	**7**	**8 (7.84%)**
**Total**	**22 (21.57%)**	**80 (78.43%)**	**102**
**Seizure types**	**Focal**	**FTBTCS**	**Both**
**Number of patients**	**101 (99.02%)**	63 (61.76%)^*^	**62 (60.78%)**
**Gender**	**Male**	**Female**	**Overall**
**Number of patients**	**58 (56.86%)**	**44 (43.14%)**	**102**
**Resection**	**Complete**	**Incomplete**	**Unclear**
**Number of patients**	**70 (68.63%)**	**13 (12.75%)**	**19 (18.63%)**
**Family history**	**Negative**	**Positive**	**Unclear**
**Number of patients**	**72 (70.59%)**	**19 (18.63%)**	**11 (10.78%)**
**Age at epilepsy onset (mean** **±** **SD)**		8.03 ± 8.08 years (SEM = 0.8, median = 6)	
**Age at surgery (mean** **±** **SD)**		25.44 ± 15.06 years (SEM = 1.49, median = 23)	
**Duration of epilepsy (mean** **±** **SD)**		17.42 ± 14.18 years (SEM = 1.40, median = 14)	

*FTBTCS, focal to bilateral tonic–clonic seizure*.

* *In 53 of these patients, FTBTCS was definitely under anticonvulsant therapy*.

We analyzed separately the longest available FU (Figure 1). After a mean long-term FU of 62.83 ± 5.06 (mean ± standard error; median 46.5) months, 55 patients (53.92%) were completely seizure free (Engel class IA), and altogether, 70 patients (68.63%) had an excellent (Engel class I) outcome [15 patients (14.71%) with Engel class IB/IC/ID, 11 (10.78%) with Engel class II, 14 (13.73%) with Engel class III, and 7 (6.86%) with Engel class IV outcome]. Compared with the outcomes at 12 months of FU for the same patients, these measures indicated a slight worsening, which reached significance (*p* < 0.01, sign test).

At 12 months of FU, 93 out of the 102 patients (91.18%) was on anticonvulsant pharmacotherapy (AED; four patients discontinued, in five cases we did not have reliable information on drug intake); at the longest available FU, 81 patients (79.41%) were taking AEDs, 21 (20.59%) already discontinued, and 32 patients (31.37%) converted already from anticonvulsant polytherapy to monotherapy.

Four patients (two with Engel class III and two with Engel class IV outcome at longest FU) underwent a second operation due to unfavorable outcome after the first surgery. Two of them became completely seizure free (Engel class IA): one of them attained Engel class II and the other patient had Engel class III outcome after the second surgery.

### Post-surgical Outcomes Over 18 Years

**Figure 2:**
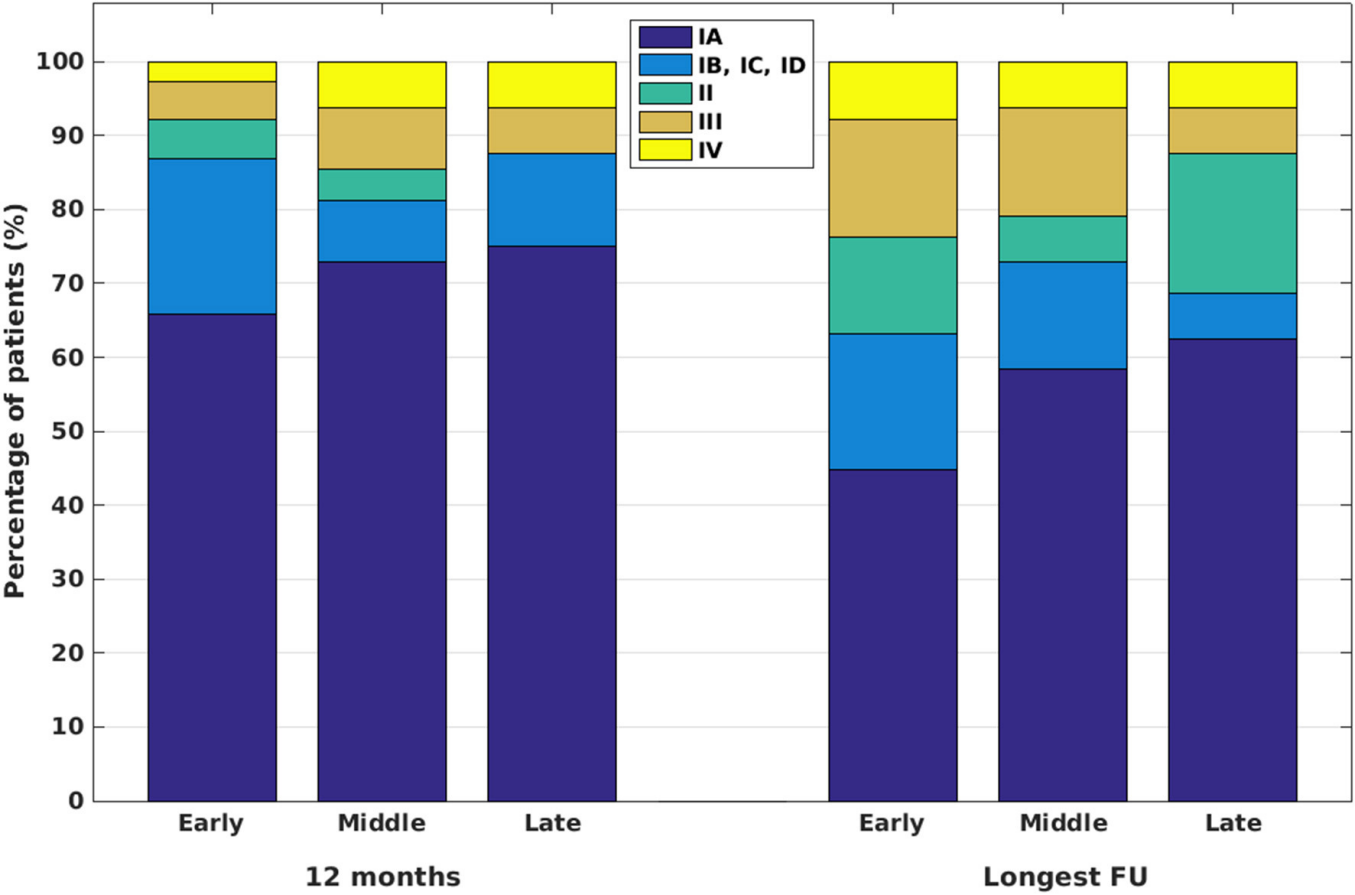
Post-surgical outcomes for patients operated on in the periods between 2002 and 2007 (“early”), between 2008 and 2013 (“middle”), and between 2014 and 2019 (“late”).

We also evaluated the possible effect of advancement in diagnostic and technical modalities on post-surgical outcomes over the last two decades. This factor can be best studied in a monocenter setting, since a number of potential confounders resulting from distinct and variable diagnostic approaches and surgical strategies over multiple epilepsy centers can be discarded. By looking at a period of 18 years, we sorted cases in three groups spanning 6 years each: *early*: operated between 2002 and 2007 (38 patients), *middle*: operated between 2008 and 2013 (48 patients), and *late*: surgery between 2014 and 2019 (16 patients) (Figure 2). Outcomes of patients operated on in different periods did not show a statistically significant difference either for 12 months or for the longest available FU (chi-square tests and Fisher's exact tests). Within-group comparisons of outcomes at 12 months and the longest FU revealed a significant difference only for the “early” group (*p* = 0.021, sign test).

### Factors Influencing Post-surgical Outcomes

**Figure 3:**
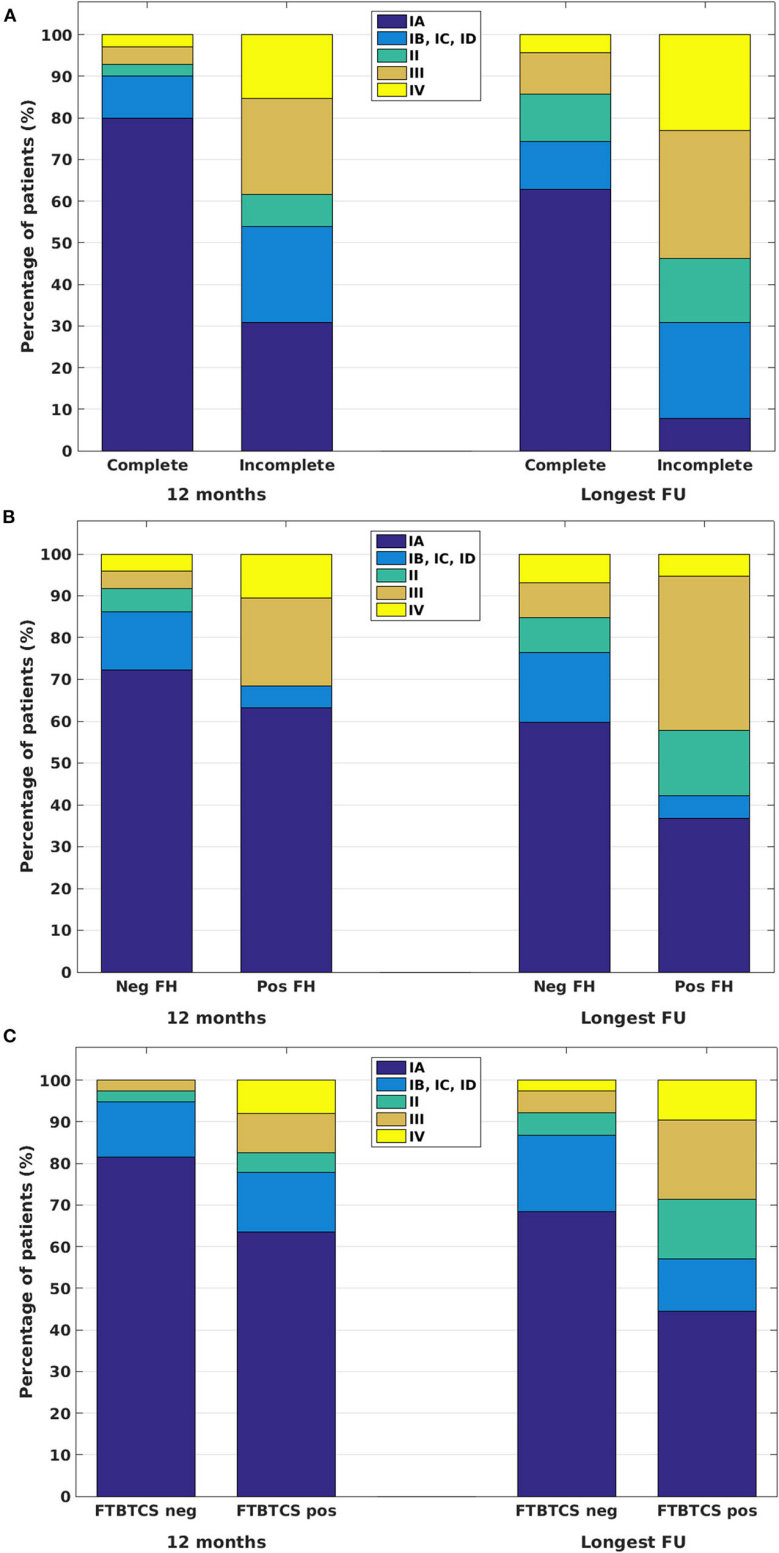
Post-surgical outcomes at 12 months and at the longest available FU in patients with complete and incomplete resections of FCD II **(A)**, for patients with negative (Neg FH) and positive (Pos FH) family history for epilepsy or epileptic seizures **(B)**, and for patients without (FTBTCS neg) and with (FTBTCS pos) focal to bilateral tonic–clonic seizures in their case history **(C)**.

We further analyzed factors possibly contributing to or determining post-surgical outcome with univariate analysis. From the examined variables [exact histopathology, lobar lesion location, history of focal to bilateral tonic–clonic seizures (FTBTCS), extent of resection, and family history for epilepsy], the extent of resection (complete vs. incomplete), history of FTBTCS, and lobar lesion location (frontal vs. non-frontal, multilobar or insular) appeared to have a significant influence on post-surgical outcome in the univariate analysis at 12 months, and the extent of resection, history of FTBTCS, and family history for epilepsy or epileptic seizures have a significant influence on post-surgical outcome at the longest available FU (Table 2; Figure 3).

Table 2Univariate analysis for possible predictors of post-surgical outcome.

**Table:** 

**Predictors**		**Histology**		**Location**		**Family history**		**Extent of resection**		**FTBTCS**	
		**12 months**	**Longest FU**	**12 months**	**Longest FU**	**12 months**	**Longest FU**	**12 months**	**Longest FU**	**12 months**	**Longest FU**
Engel IA, IB–IC–ID, II, III, IV	Chi-square test	0.22	**0.02**	**0.03**	0.73	0.07	**0.02**	** <0.01**	** <0.01**	0.20	0.05
Engel I vs. Engel II–III–IV	Chi-square test	0.72	0.28	** <0.01**	0.29	0.07	** <0.01**	** <0.001**	** <0.01**	**0.02**	** <0.01**
	Fisher's test	0.74	0.31	** <0.01**	0.38	0.09	**0.01**	** <0.01**	** <0.01**	**0.03**	** <0.01**
Engel I–II vs. Engel III–IV	Chi-square test	0.29	**0.04**	** <0.01**	0.23	** <0.01**	**0.01**	** <0.01**	** <0.01**	**0.03**	**0.01**
	Fisher's test	0.28	0.07	** <0.01**	0.31	**0.02**	**0.02**	** <0.01**	** <0.01**	**0.03**	**0.02**

**Table:** 

		**IIA**	**IIB**	**IIA**	**IIB**	**Fron**.	**Nonf**.	**Fron**.	**Nonf**.	**Neg**.	**Pos**.	**Neg**.	**Pos**.	**Com**.	**Inc**.	**Com**.	**Inc**.	**No**	**Yes**	**No**	**Yes**
Outcomes (Engel)	IA	68.18	71.25	45.45	56.25	80	54.06	58.46	45.95	72.22	63.16	59.72	36.84	80	30.77	62.86	7.69	81.58	63.49	68.42	44.44
Percentage of patients (%)	IB, IC, ID	13.64	13.75	13.64	15	12.31	16.22	13.85	16.22	13.89	5.26	16.67	5.26	10	23.08	11.43	23.08	13.16	14.29	18.42	12.70
	II	0	5	4.55	12.50	3.08	5.41	10.77	10.81	5.56	0	8.33	15.79	2.86	7.69	11.43	15.38	2.63	4.76	5.26	14.29
	III	4.55	7.5	13.64	13.75	3.08	13.51	10.77	18.92	4.17	21.05	8.33	36.84	4.29	23.08	10	30.77	2.63	9.52	5.26	19.05
	IV	13.64	2.5	22.73	2.5	1.54	10.81	6.15	8.11	4.17	10.53	6.94	5.26	2.86	15.38	4.29	23.08	0	7.94	2.63	9.52

*FTBTCS, focal to bilateral tonic–clonic seizure; FU, follow-up; IIA, FCD type IIA; IIB, FCD type IIB; Fron., frontal; Nonf., non-frontal, multilobar or insular; Neg., negative; Pos., positive; Com., complete; Inc., incomplete*.

*Results are represented by p-values of chi-square tests (for Engel classes IA, IB–IC–ID, II, III, and IV as categories) and by p-values of chi-square tests and Fisher's exact tests comparing excellent with non-excellent (Engel class I vs. Engel classes II–III–IV), and favorable with unfavorable outcomes (Engel classes I–II vs. Engel classes III–IV). In the lower panels, the percentage of patients with respective outcomes is listed separately. Bold values indicate significant differences*.

We further analyzed the above-discussed and additional factors (age at epilepsy onset, age at surgery, duration of epilepsy) with multivariate logistic regression analysis. The stepwise regression analysis verified several, partially interacting factors (age at epilepsy onset, duration of epilepsy, age at surgery, extent of resection, and lesion location) determining outcomes at 12 months, of which the extent of resection and lesion location appeared to be the most relevant (Table 3). The analysis revealed completeness of resection and history of FTBTCS as significant independent predictors for long-term outcome (Table 3). The family history for epilepsy or epileptic seizures displayed significant values only for long-term outcomes and only by contrasting Engel class I against Engel class II–IV outcomes.

**Table 3:** Multivariate logistic regression analysis for possible predictors of post-surgical outcome: *p*-values for predictor variables.

	**12 months**			**Longest FU**		
**Outcome classes**	**Engel IA, IB–D, II, III, IV**	**Engel I vs. II–IV**	**Engel I–II vs. III–IV**	**Engel IA, IB–D, II, III, IV**	**Engel I vs. II–IV**	**Engel I–II vs. III–IV**
**Stepwise regression**						
Predictors	Age at onset *p* < 0.0001 Duration of epilepsy *p* < 0.01 Lesion location *p* = 0.02 Extent of resection *p* = 0.49 Duration of epilepsy* Extent of resection *p* = 0.02	Age at onset *p* < 0.01 Duration of epilepsy *p* < 0.05 Lesion location *p* = 0.02 Extent of resection *p* < 0.001	Age at surgery *p* < 0.001 Extent of resection *p* < 0.001 Lesion location *p* = 0.36 Age at surgery* Lesion location *p* < 0.01	Extent of resection *p* < 0.0001 FTBTCS *p* < 0.01	Extent of resection *p* < 0.001 FTBTCS *p* < 0.01 Family history *p* = 0.01	Extent of resection *p* < 0.001 FTBTCS *p* = 0.02

Interacting variables are marked with*.

*FTBTCS, focal to bilateral tonic–clonic seizure; FU, follow-up*.

### Positive Family History in Patients With Glioneuronal Tumors

To elucidate the potential influence of positive family history for epilepsy or epileptic seizures on post-surgical outcome, we also analyzed data in a larger set of patients operated on with glioneuronal tumors over the same period in our center. Out of 94 operated patients, 8 (8.51%) displayed positive family history for epilepsy; in 73 patients, family history was negative; and in 13 patients, we did not have reliable information on family history. Interestingly, all 8 patients were completely seizure free both at 12 months and at the longest available FU; among patients with negative family history, 70.59 and 54.79% (12 months and longest FU, respectively) were completely seizure free (Engel class IA). Outcome differences between the positive and negative family history groups with glioneuronal tumors were not found to be statistically significant (univariate analysis with chi-square tests and Fisher's exact test). When comparing patients with positive family history from the FCD group and from the glioneuronal tumor group, outcome differences were not significant at 12 months, but these were significant for the longest FU, when we contrasted excellent (Engel class I) to non-excellent (Engel classes II–IV) outcomes (*p* = 0.0052, chi-square test; *p* = 0.0082, Fisher's exact test). By contrasting favorable (Engel classes I–II) to unfavorable (Engel III–IV) outcomes, the chi-square test indicated a significant difference (*p* = 0.03), which was not supported by Fisher's test (*p* = 0.06).

## Discussion

Here, we report post-surgical outcomes in a series of 102 patients who underwent epilepsy surgery for medically refractory epilepsy related to FCD II. Concordant with the literature (10–12, 16–22), the majority of our patients displayed an excellent or at least favorable outcome both at 12 months and at the longest available (median 46.5 months) FU. Moreover, the extent (completeness) of resection and the absence of FTBTCS in the patient history were found to be important predictors for long-term positive post-surgical outcome, whereas age at epilepsy onset, age at surgery, duration of epilepsy, exact histopathology (FCD type IIA vs. IIB), and lesion location (frontal vs. non-frontal, multilobar or insular) did not have a consistently significant effect on long-term outcomes after epilepsy surgery. Even though positive family history for epilepsy or epileptic seizures was associated with a trend toward worse outcome at the longest available FU, this effect did not turn out to be consistently significant in the multivariate analysis. Outcomes after epilepsy surgery did not show a remarkable difference over the last 20 years in our center.

Outcome measures showed a slight but significant worsening tendency at long-term compared with 12 months of FU. Furthermore, the long-term seizure freedom rates might be underestimated when investigating patient collectives with late FU in an outpatient setting, where patients not attaining seizure freedom are probably overrepresented and patients reaching long-term seizure freedom are underrepresented for not having the need to seek further medical care. For that reason, on the other hand, we performed a detailed telephone interview with patients not having contact with our clinic during the 12 months preceding data acquisition for the study. Nevertheless, since some of these patients could not be reached any more (changing address, etc.), and some of them did not provide a written informed consent for the phone interview, we cannot completely rule out that the slight worsening tendency—though reported in several studies (35)—partially resulted from a disequilibrium of patients as described above.

The observation that the extent (i.e., completeness) of resection had a significant influence on post-surgical outcome is in concordance with previous publications (10, 12, 21, 35–37).

Even though long-term outcomes were slightly worse in patients with positive family history for epilepsy or epileptic seizures than in those with negative family history, this factor did not consistently reach significance in the multivariate regression analysis. On the other hand, we cannot rule out that the relatively low sample size of patients with positive family history (19 patients) did not allow the identification of a statistically significant effect, only a trend. Certainly, we cannot exclude that some of the patients with a positive family history for epilepsy might have had genetic variants in susceptibility genes independent of the FCD. On the other hand, our results are also reconcilable with recent observations that focal cortical malformations can be operated with excellent post-surgical outcome even in case of justified genetic alterations, especially affecting the mTOR pathway (38–41).

To further clarify the potential role of positive family history for epilepsy on post-surgical outcome, we compared data from patients operated on with glioneuronal tumors during the same period. Apparently, positive family history for epilepsy did not exert a significant influence on post-surgical outcomes among patients with glioneuronal tumors in a univariate analysis, and patients with positive family history and glioneuronal tumors displayed in certain measures a better outcome than those with FCD II. As the sample sizes were low, however, one needs to interpret the observed effects with caution. On the other hand, the observed difference between patients with FCD and glioneuronal tumors and with positive family history for epilepsy might suggest a possible FCD-specific effect in determining post-surgical outcomes.

The finding that history of FTBTCS in the patient history can be regarded as a negative predictor might hypothetically be related to a more widespread epileptogenic network reaching far beyond the histological boundaries of FCD in many patients with FTBTCS.

The effect of precise histopathology of FCD is somewhat controversial in the literature. Although several publications do not present relevant differences between distinct histopathological subtypes [(42): FCD type I vs. II; (16): FCD types I, II, and III; (12): FCD type I vs. II] or find non-significant trends [(11): FCD IIB slightly better than IIA], further studies found either better results in FCD type I (19) or in FCD type II (20, 23) and better outcomes in FCD type IIB than in type IIA (43). The effect of associated lesions in terms of a dual pathology also seems to be controversial. Patients operated on with glioneuronal tumors or hippocampal sclerosis associated with FCD type I were reported to have a significantly better post-surgical outcome (24), whereas another investigation found worse outcomes in FCD type III compared with FCD types I and II (44). In our series, we had a disproportionate distribution between FCD IIA and IIB, meaning a potential limitation in our results. This misbalance is most probably related to the better MR diagnostic features of FCD IIB compared with IIA (11) and also to the fact that patients with lesional MRI preferentially undergo epilepsy surgery in our center. In addition, we also excluded cases with dual pathologies and FCD types I and III in order to have more homogeneous histological groups for the analysis.

The precise location of FCD may also have an impact on post-surgical outcome. Among pediatric patients, frontal or temporal FCD location was associated with a significantly better outcome compared with central or posterior locations (12). A temporal lobe location in patients with FCD IIA was also found to be a predictor for positive outcome (35). In our series, we did not find a consistent and significant difference between long-term outcomes in frontal vs. non-frontal location; on the other hand, due to the paucity of temporal lobe location, we could not examine this lobar location separately.

The literature also reports conflicting results on the influence of early epilepsy surgery on post-surgical outcome. For instance, in a series of pediatric patients, age at surgery (in infancy vs. at later age) did not exert a significant influence on post-surgical outcome (12). In a series reporting on patients operated on with FCD type II before and after 20 years of age, a statistically significant difference in post-surgical outcomes was not reported either (45). However, effects can be confounded by other variables and also depend on the compared patient populations. In the pediatric series from the Freiburg Epilepsy Center, for instance, patients operated at younger ages more frequently underwent extensive operations (i.e., multilobar resections and hemispherectomies) than those operated at a later age; nevertheless, they displayed worse outcomes (42). Moreover, in the complete series from Freiburg comprising both adults and children, early surgery (before 18 years of age) proved to be a positive predictor for optimal outcome (16). In the current series, we did not find a hint on the significant effect of age at epilepsy onset, age at epilepsy surgery, and duration of epilepsy on long-term post-surgical outcomes.

The strength of our investigation relies on the big sample size and the length of FUs. Besides, the effect of family history for epilepsy or epileptic seizures on post-surgical outcome—to our best knowledge—has not been systematically analyzed in larger patient groups with FCD so far. A certain limitation may be given by a misbalance between the sample size of the reported FCD subtypes (FCD IIA and IIB), the paucity of less frequent (especially temporal and occipital) lesion locations, and the retrospective monocenter design.

Epilepsy in early childhood is frequently associated with relevant cognitive and developmental disturbances (46, 47), and epilepsy surgery early in the course of the disease can have an immense impact on the development of affected individuals (47). Concordant with published results on bigger patient collectives, epilepsy surgery still tends to be relatively late compared with epilepsy onset (48) despite diagnostic advancements during the last decades (31–33). In the analyzed group, the median duration of epilepsy till surgery was 14 years, meaning that for certain patients, it took several decades to access this therapeutic modality.

## Conclusions

Post-surgical seizure control in patients with FCD II is slightly worse at the longest available FU with a median of 46.5 months compared with 12 months. Complete lesion resection and the absence of FTBTCS in the patients' history are found to be the most important predictors for optimal long-term post-surgical outcome, whereas exact histopathology (FCD IIA vs. FCD IIB), lobar lesion location, age at epilepsy onset, age at surgery, and epilepsy duration do not have a significant impact on long-term post-surgical outcome. Positive family history for epilepsy might have a marginal effect on post-surgical outcome. This result also supports the view that “heritable” focal cortical malformations can be excellent subjects for epilepsy surgery.

## Data Availability Statement

The raw data supporting the conclusions of this article will be made available by the authors, without undue reservation.

## Ethics Statement

The studies involving human participants were reviewed and approved by Ethik-Kommission - Medizinische Fakultät Bonn. Written informed consent from the participants' legal guardian/next of kin was not required to participate in this study in accordance with the national legislation and the institutional requirements.

## Author Contributions

AR and CE contributed to conception and design of the study. AR, AB, and CQ collected the data. AR and AB analyzed the data. AR wrote the first draft of the manuscript. AB, CQ, VB, HV, RS, and CE wrote sections of the manuscript. All authors contributed to manuscript revision, read, and approved the submitted version.

## Conflict of Interest

The authors declare that the research was conducted in the absence of any commercial or financial relationships that could be construed as a potential conflict of interest.

## Abbreviations

AED: antiepileptic drug
FCD: focal cortical dysplasia
FTBTCS: focal to bilateral tonic–clonic seizure
FU: follow-up
GABA: gamma amino butyric acid
ILAE: International League Against Epilepsy
MRI: magnetic resonance imaging
PET: positron emission tomography
SPECT: single-photon emission computed tomography.
